# Research on Multiaxial Random Vibration Fatigue Assessment Method for Vehicle-Mounted Equipment Based on IEC 61373 Standard

**DOI:** 10.3390/ma19071450

**Published:** 2026-04-04

**Authors:** Zhixiang Luo, Chengrui Guang, Yi Liu, Zhongcheng Hu, Ji Fang

**Affiliations:** 1 State Key Laboratory of Heavy-Duty and Express High-Power Electric Locomotive, Zhuzhou 412001, China; 2 Zhan Tianyou College (CRRC College), Dalian Jiaotong University, Dalian 116028, China; g921593798@163.com (C.G.); liu_yi0826@163.com (Y.L.); huzhongcheng1130@163.com (Z.H.)

**Keywords:** multiaxial, IEC 61373 standard, random vibration, vehicle-mounted equipment, fatigue assessment

## Abstract

At present, most of the research methods for vibration fatigue of welded structures mainly focus on uniaxial stress, ignoring the influence of shear stress. To this end, by combining the ASME structural stress method with the random and vibration analysis theory outlined in the IEC 61373 standard, a new method for evaluating the fatigue life of multi-axis random vibration problems in the frequency domain has been proposed. This method extends the structural stress method to multi-axis scenarios to accurately extract the local multi-axis structural stress state at the weld toe. Its advantage lies in the fact that it not only accounts for the influence of load frequency distribution and structural modal vibrations on fatigue life, but also incorporates the effect of local multiaxial stress conditions in the weld on fatigue life. Additionally, it includes corrections for non-proportional multiaxial stress conditions, resulting in fatigue assessment results that more closely reflect actual conditions. It was validated by comparing the local multiaxial stress, phase difference between shear and normal stress, and equivalent structural stress power spectrum of 0° and 30° fillet welded specimens with test results. Subsequently, it was applied to a multiaxial random vibration fatigue assessment of a vehicle-mounted electrical cabinet with experimental verification. The results indicate that fatigue life estimates based on a multi-axis stress state are lower than those obtained using a uniaxial method. Compared to traditional uniaxial methods, the multi-axis fatigue life estimates show a significant reduction ranging from 4.20% to 88.35%, effectively accounting for damage caused by shear stress. The fatigue assessment results are more closely aligned with experimental data, thereby validating the effectiveness of the proposed new method. The frequency-domain multiaxial random vibration fatigue assessment method proposed in this article provides a new technology for the design and evaluation of welded structures of vehicle-mounted equipment in rail vehicles. This method reduces costs during the design phase of rail vehicles, offering positive economic implications.

## 1. Introduction

With the continuous development of high-speed and lightweight rail vehicles, increased train operating speeds have amplified high-frequency load components. Modal frequencies of vehicle-mounted equipment have decreased due to lightweight design, intensifying structural vibrations and making fatigue failure of welds in Vehicle-mounted equipment an increasingly prominent issue. Therefore, evaluating the fatigue reliability of vehicle-mounted equipment welds under vibrational conditions is now the foremost priority in the structural design process for installing such equipment.

The fatigue assessment of rail vehicle welded structures has evolved from traditional nominal stress methods to localized, high-precision mechanical models. In the early stages, when the focus was on engineering practicality, the structural stress method proposed by Dong et al. [1] demonstrated exceptional reliability in predicting fatigue life under quasi-static loads due to its outstanding mesh insensitivity. To accommodate the complex dynamic operating environment of rail vehicles, Fang et al. [2] and Li et al. [3] successfully extended this theory to the frequency domain and modal space, establishing an efficient evaluation framework for the frequency-domain structural stress method. However, when faced with complex random vibrations under real-world operating conditions, this evaluation method which assumes uniaxial loading gradually revealed its limitations. Although researchers such as Jin et al. [4] have employed simplified dynamic stress spectra, and Nie et al. [5] have explored engineering rapid assessment schemes based on Steinberg’s three-zone method, these methods often fail to meet the requirements of high-reliability design in terms of prediction accuracy when dealing with complex damage induced by structural spatial modes, due to the inherent limitations of the uniaxial theory.

To overcome the accuracy limitations of single-axis theory, researchers have begun to delve deeper into microstructural evolution and macroscopic multi-axis time-domain loading mechanisms. At the microstructural level, the microstructural heterogeneity and grain coarsening in the heat-affected zone (HAZ) of welds can lead to severe strain localization under complex stresses. Studies by Zhang et al. [6] and Karabulut et al. [7] have all confirmed that these microstructural changes profoundly modulate macroscopic fatigue behavior. It is worth noting that modern research indicates that improving the microstructural integrity of phase boundaries can directly influence the dynamic response of power transmission systems. For example, Bukvić et al. [8] demonstrated that introducing nano-additives such as carbon nanotubes (CNTs) into lubricants can strengthen contact interfaces at the microscopic physical level, thereby significantly reducing mechanical vibration and friction losses. In the fatigue assessment of macroscopic passive load-bearing structures, Cao et al. [9], Bufalari et al. [10] and Pezeshki et al. [11] proposed multiaxial damage parameters capable of accurately handling complex stress interactions by incorporating non-proportional load paths. However, as pointed out in the studies by Zha et al. [12] and Joo et al. [13], the extremely high computational complexity makes it difficult to directly apply these methods to rapid evaluations during the preliminary design phase of large-scale rail vehicles. This complexity stems from either the high-precision characterization of micro-scale interfaces or multi-axis tracking calculations based on massive time-domain data.

This has placed current research in a dilemma: high-precision multiaxial and microstructural model calculations are prohibitively expensive, while efficient frequency-domain structural stress methods suffer from theoretical blind spots. To strike a balance between the two, a number of innovative frequency-domain correction techniques have recently emerged. The Multi-Input Fatigue Damage Spectrum (MI-FDS) proposed by Proner et al. [14] and the FDmS spectrum developed by Aimé et al. [15] attempt to compensate for computational errors arising from complex interactions by introducing cross-spectral density (CSD); Palmieri et al. [16], meanwhile, utilize modal decomposition to accelerate the evaluation of non-Gaussian loads. However, as Ales et al. [17] emphasized in their review, traditional frequency-domain methods still face significant challenges when addressing multiaxial fatigue problems. The core reason lies in the fact that the mathematical process of converting time-domain signals to power spectral density (PSD) irreversibly loses the coupling information between different stress components.

For uniaxial fatigue, mature life prediction methods are now available in both time and frequency domains [18]. However, research on multiaxial fatigue has primarily focused on the material level or simple loading conditions, with relatively limited studies on multiaxial fatigue under random loading. In engineering applications, fatigue crack propagation in welds is typically dominated by normal stress perpendicular to the weld direction, with shear stress often neglected. However, under complex structural configurations and loading patterns, shear stress significantly increases, making their impact on weld fatigue life critical. When shear stress is linearly proportional to normal stress, their influence is relatively straightforward; yet non-proportional relationships between the two stresses can induce additional damage effects [19].

Currently, the fatigue-resistant design of on-board equipment for rail transit primarily relies on standards such as IEC 61373-2010 [20] and GB/T 21563-2018 [21], which specify the test conditions and requirements for random vibration fatigue testing of on-board equipment. However, translating these standard inputs into accurate multi-axis fatigue life predictions for specific welds remains a challenge. This paper combines the IEC 61373 standard with the ASME structural stress method to propose a multi-axis random vibration fatigue assessment method suitable for on-board equipment in rail vehicles. Unlike existing studies that rely on traditional uniaxis frequency-domain analysis or require extremely computationally intensive time-domain multi-axis assessments, this study proposes a more practical frequency-domain algorithm capable of directly extracting and correcting the phase difference between normal stress and shear stress within the frequency domain. The specific significance of this paper lies in its ability to quantitatively assess non-proportional multi-axis damage caused by structural modal resonance under standard broadband excitation. By transforming complex multiaxis fatigue mechanics into a practical engineering workflow, this method enables designers to identify high-risk weld regions during the early stages of numerical simulation. Ultimately, it effectively addresses the shortcomings of traditional uniaxis methods, which tend to underestimate localized fatigue damage, and provides a robust, cost-effective, and efficient research method for the safety assessment and weight-reduction optimization of vehicle-mounted equipment.

## 2. Fatigue Assessment Method for Welded Structures

**Figure 1:**
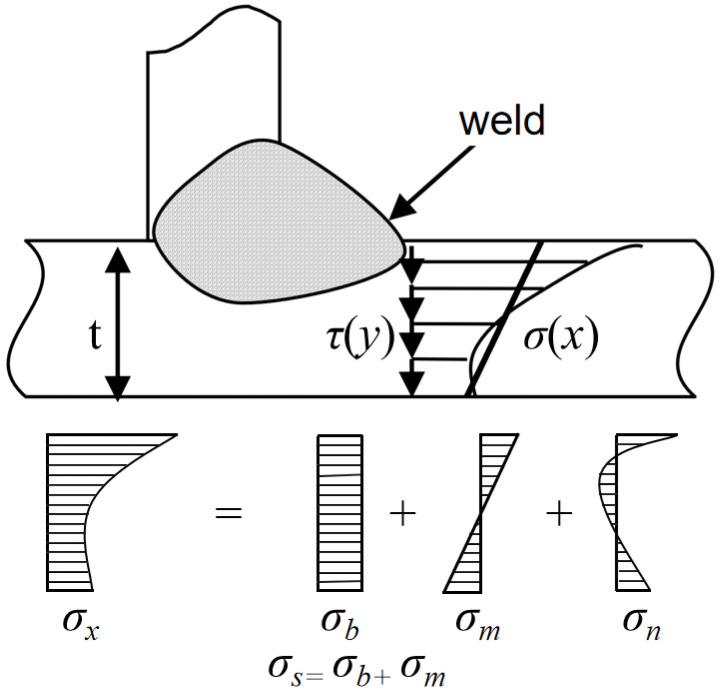
The stress distribution and structural stress of the weld toe of definition.

The structural stress method has gained widespread application in the engineering community due to its clear conceptual framework and high precision in assessing the fatigue life of welded structures [22]. Structural stress is the primary parameter in the main S-N curve equation and plays a crucial role in assessing the fatigue life of welded structures. The structural stress value equals the sum of membrane stress $\sigma_{s}$ and bending stress $\sigma_{b}$ (Figure 1). $\sigma_{x}$ represents the total stress at the weld toe, while $\sigma_{n}$ denotes the self-balancing residual stress, the influence of which has been accounted for in the experiments.

**Figure 2:**
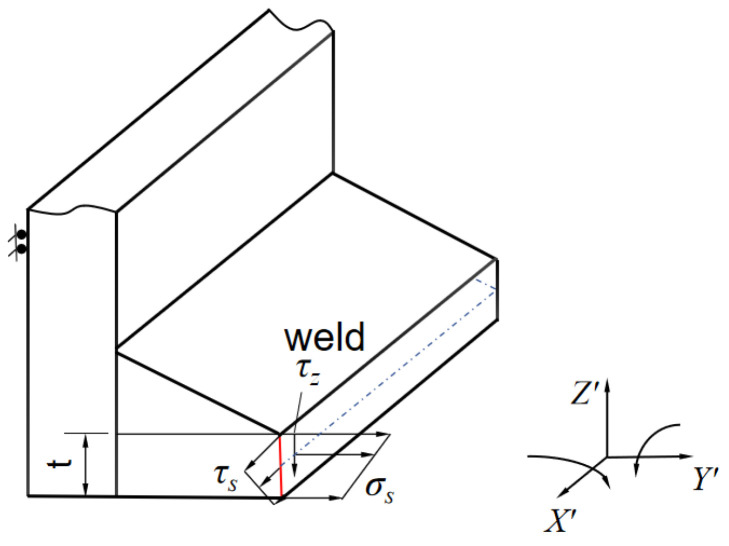
The definition of the three-dimensional stress of the welded components.

Rail vehicle structures are predominantly fabricated from thin plate weldments. During operational service, the stress state at welds typically exhibits three-dimensional characteristics. Using the common weld penetration depth as the local coordinate system, the definition of three-dimensional structural stress components acting on the shear plane traversing the thickness direction at the weld location is illustrated in Figure 2. Normal stress $\sigma_{s}$ is perpendicular to the weld line and is the key parameter responsible for the opening-type failure mode. In-plane shear stress $\tau_{s}$ acts along the weld’s longitudinal direction (parallel to the weld line) and represents the longitudinal shear force acting on the weld. Transverse shear stress $\tau_{z}$ acts along the plate thickness direction (perpendicular to the plate surface) and represents the transverse tearing force acting on the weld. In engineering structures, from the perspective of fracture mechanics, the stress state corresponds to a Type I (opening) crack. The normal stress perpendicular to the weld direction is the primary factor driving fatigue crack propagation; that is, the direction of the external load is perpendicular to the crack surface, causing microscopic defects at the weld toe to open along the plate thickness and gradually develop into macroscopic cracks, while shear stresses $\tau_{s}$ and $\tau_{z}$ are generally small and can be neglected [23].

When a structure is subjected to shear and normal loads applied proportionally, the stress components vary proportionally over time. The corresponding principal stress directions remain constant, with peaks and troughs occurring simultaneously. Therefore, the equivalent structural stress range can be directly determined by the variation range of the stress components. When in-plane shear stress $\tau_{s}$ is considered, the normal structural stress range ${\Delta \sigma }_{s}$ and the in-plane shear stress range ${\Delta \tau }_{s}$ can be combined into an combined structural stress range ${\Delta \sigma }_{e0}$ via Equation (1). Simultaneously, the rainflow counting method can still track the loading time history. However, if stress components vary independently or exhibit phase differences over time, the impact of non-proportional effects on fatigue damage must be considered. The physical significance of the constant *β* in Equation (1) is the ratio of fatigue strength between normal and shear stress based on fatigue testing.

(1) $$\Delta \sigma_{e0}=\sqrt{{\left(\;\Delta \sigma_{s}\right)}^{2}+\beta {\left(\Delta \tau_{s}\right)}^{2}}$$

In the double-logarithmic coordinate system based on the S-N curve, since the two sets of data may not be mutually parallel, the *β* value can be determined using the average value beyond the life range. Analysis of a series of multiaxial fatigue test data indicates that the *β* values for steel–aluminum alloy welded joints typically vary between 3 and 4. Test data suggest that *β* = 3 is applicable for steel [24]. In the structural stress calculation, the coefficient *β* reflects the relative sensitivity of the material’s fatigue life to shear stress versus normal stress, which is highly dependent on material properties and loading conditions. For ductile materials such as steel evaluated in this study under multiaxial random vibration loads, microscopic fatigue damage is primarily driven by plastic distortion energy. According to the von Mises equivalent stress criterion and relevant ASME fatigue evaluation standards, the ratio of normal to shear fatigue strength for ductile materials is approximately $\sqrt{3}$. Consequently, adopting *β* = 3 in the structural stress calculation is physically justified and aligns with established engineering codes [25].

According to the provisions for correcting multiaxial fatigue in structural stress methods under Chapter 5 of Volume VIII, Book 2 of the ASME Code: When the range of structural shear stress is non-negligible (∆τ>∆σ/3), the equivalent structural stress range must be corrected [26]. After removing the plate thickness correction and load ratio correction factors from the multiaxial equivalent structural stress calculation formula in the ASME standard, the following non-proportional loading correction multiaxial composite structural stress calculation formula was derived (Equation (2)):

(2) $$\Delta \sigma_{e}=\frac{1}{F(\delta )}\sqrt{{\left(\;\Delta \sigma_{s}\right)}^{2}+3{\left(\Delta \tau_{s}\right)}^{2}}$$

where *δ* represents the phase difference between the structural stress perpendicular to the weld direction and the shear structural stress parallel to the weld direction. F(δ) is the multiaxial correction factor for normal stress and shear stress, with the range ($1/\sqrt{2}$,1). This function accounts for the combined effects of normal stress and shear stress by adjusting the amplitude of equivalent structural stress through the phase angle δ. For convenience, we have simplified 1/F(δ) linearly (Equation (3)).

(3) $$\frac{1}{F(\delta )}=1+\frac{2(\sqrt{2}-1)}{\pi }\delta$$

**Figure 3:**
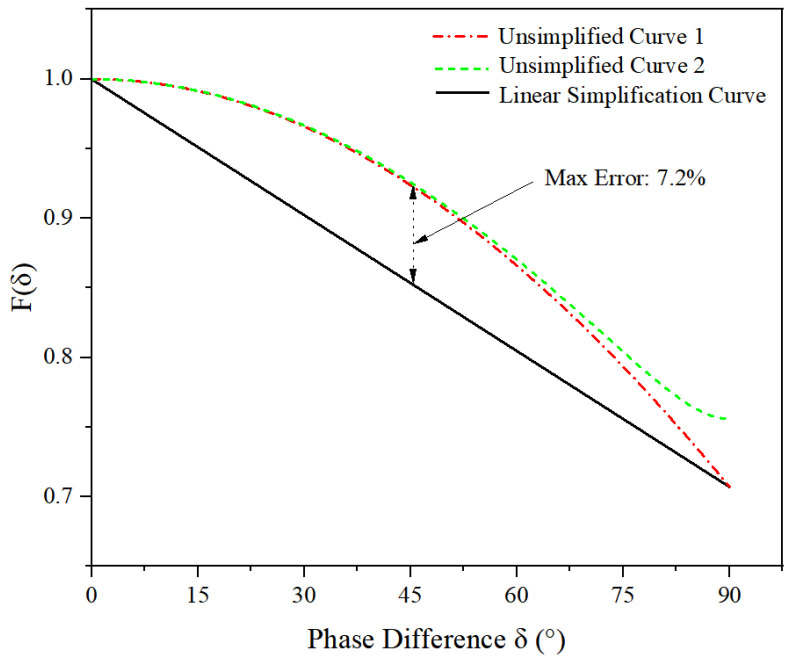
Comparison of Linear and Non-Linear Simplifications.

Regarding the potential errors introduced by linear simplification, the following explanation is provided: The ASME standard provides a fitting formula for the multi-axis correction factor F(δ); however, because this formula is overly complex—involving various combinations of normal and shear stress magnitudes—it significantly increases the complexity of subsequent calculations. Figure 3 presents the F(δ) curves for two typical operating conditions under the standard-specified sinusoidal load, corresponding to normal stress-to-shear stress ratios of 2 and $\sqrt{3}$, respectively, together with their linearized fitting curves. Calculations indicate that the maximum error resulting from this linear simplification is approximately 7.2%. It should be noted that the F(δ) values obtained from this linear simplification are generally lower than the standard values; as shown in Equation (2), F(δ) is inversely proportional to the combined structural stress range ${\Delta \sigma }_{e0}$. This means that this simplification yields more stringent stress assessment results, thereby ensuring the conservativeness of the assessment.

Under simple cyclic loading, the phase angle in multiaxial correction formulas is relatively easy to obtain; however, under complex random loading, the phase angle between the shear and normal stress is difficult to determine. In the frequency-domain structural stress method, structural stress is represented by power spectra, which lack phase difference information, limiting the application of the aforementioned multiaxial correction formulas in this method.

## 3. Multiaxial Fatigue Assessment Method for Welded Structures Based on IEC 61373 Standard

### 3.1. Method of Muitiaxial Random Vibration Fatigue Testing

**Figure 4:**
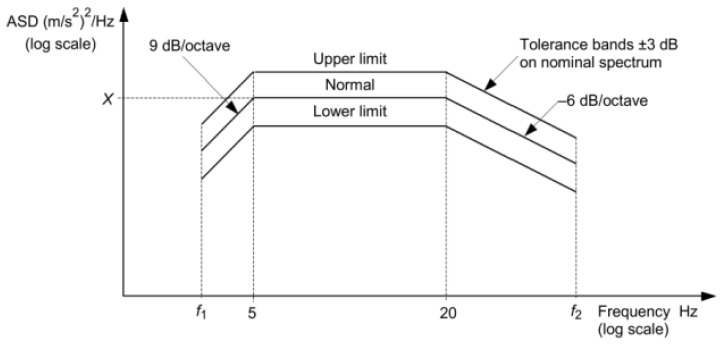
Schematic diagram of random vibration acceleration power spectrum in IEC 61373-2010 standard.

Vehicle-mounted equipment for rail vehicles should undergo vibration fatigue testing based on random loads specified in IEC 61373 or EG standard to verify fatigue resistance. IEC 61373-2010 [20] categorizes equipment by mounting location into three classes: axle-mounted (Class 3), frame-mounted (Class 2) and body-mounted (Class 1, subdivided into 1A and 1B). Tests shall be conducted by applying separate loads in three directions according to the power spectrum and grade parameters shown in Figure 4, each undergoing a 5-h simulated long-life random vibration fatigue test. If vehicle-mounted equipment does not exhibit fatigue failure after 15 h of testing, it is deemed to meet design requirements.

To perform random vibration fatigue analysis on critical structural welds during the design phase, this paper employs the structural stress method from the ASME standard. Based on the proposed frequency-domain structural stress method, the authors derive a linear relationship between structural stress perpendicular to the weld line direction and the applied load under linear systems [2].

First, we perform a Fourier transform on the general equation of structural dynamics 4 to obtain the Equation (5) in the frequency domain.

(4) $$M\ddot{x}+C\dot{x}+Kx=f(t)$$

(5) $$\left[-\omega^{2}M+j\omega C+K\right]u(\omega )=p(\omega )$$

In the equation, *M* represents the mass matrix, *K* represents the stiffness matrix, and *C* represents the damping matrix. If none of these vary with time, the system is classified as a linear vibration system. Typically, the structural vibration system of a rail vehicle can be approximated as a linear vibration system. When an acceleration excitation is applied, the expression in the frequency domain after a Fourier transform is as Equation (6):

(6) $$\ddot{u}(t)=-\omega^{2}U\cdot e^{j\omega t}$$

Assuming the external load is a unit frequency-domain load, the linear frequency response function of the node displacement at the weld toe of the welded structure can be calculated using Equation (7) as follows:

(7) $$H(\omega )=\frac{1}{K-\omega^{2}M+j\omega C}$$

After calculating the node forces in the frequency domain, performing coordinate transformations, and computing the equivalent line forces and line moments, the real-part expression of the frequency response function for the structural stresses at each node of the welded structure can be obtained using the structural stress formula as Equation (8):

(8) $$H_{\sigma }^{a}(\omega )=\sigma_{m}(\omega )+\sigma_{b}(\omega )=\frac{f_{a}(\omega )}{t}+\frac{6m_{a}(\omega )}{t^{2}}$$

Substituting the expressions for the equivalent normal force and moment into the equation yields the following complete expression for the real part and imaginary part of the structural stress frequency response function (Equation (9)):

(9) $$\left\{\begin{array}{l} H_{\sigma }^{a}(\omega )=\left\{TNB^{T}K^{e}BL^{-1}A\right\}H_{a}(\omega )\\ H_{\sigma }^{b}(\omega )=\left\{TNB^{T}K^{e}BL^{-1}A\right\}H_{b}(\omega ) \end{array}\right.$$

where *K^e^* is the stiffness matrix in the element local coordinate system; *B* is the transformation matrix from the element local coordinate system to the system coordinate system; *N* is the node force and moment synthesis matrix, enabling the combination of node forces from adjacent elements; *T* is the transformation matrix from the system coordinate system to the weld local coordinate system; *L* is the length equivalent matrix; and *A* is the substitution matrix related to plate thickness *t*. *K^e^*, *B*, *N*, *T*, *L*, and *A* are constant coefficient matrices related to structural node coordinates and plate thickness *t*; this demonstrates that there is a linear relationship between structural stress in the frequency domain and the load at a specific frequency. Therefore, we can use theories related to random vibrations to calculate the structural stress response at the welded joints under random loads. We denote this matrix of constant coefficients by *Q* (Equation (10)):

(10) $$Q=\left\{TNB^{T}K^{e}BL^{-1}A\right\}$$

The structural stress frequency response function $H_{\sigma }(\omega )$ perpendicular to the weld line direction exhibits the following linear relationship with the displacement frequency response function $H_{d}(\omega )$ (Equation (11)):

(11) $$H_{\sigma }(\omega )=Q_{1}H_{d}(\omega )$$

The structural stress power spectrum can be calculated using the following Equation (12):

(12) $$S_{\sigma }(\omega )={\left|H_{\sigma s}(\omega )\right|}^{2}S_{x}(\omega )$$

In the equation, $H_{\sigma s}(\omega )$ represents the input power spectral density. For shear stress parallel to the weld line direction, the structural stress frequency response function and its linear relationship with load can similarly be derived. The structural stress power spectrum formula is given by Equations (13) and (14).

(13) $$H_{\tau }(\omega )=Q_{2}H_{d}(\omega )$$

(14) $$S_{\tau }(\omega )={\left|H_{\sigma s}(\omega )\right|}^{2}\;S_{x}(\omega )$$

In the theoretical framework established above, $H_{\sigma }(\omega )$ and $H_{\tau }(\omega )$ clearly serve as the core transfer functions for transitioning from macroscopic system vibrations to local structural stress. By using the constant coefficient matrices *Q*_1_ and *Q*_2_ related to the geometric shape, the global dynamic displacement caused by system vibrations is linearly mapped to the local multiaxial stress state at the weld toe. This mathematical transformation seamlessly connects the global random vibration input with the local stress power spectrum. The difference between *Q*_1_ and *Q*_2_ lies in the value of matrix *N*. For the direction perpendicular to the weld, *N* is the sum of the nodal forces perpendicular to the weld and the nodal moments about the axis parallel to the weld direction. For the direction parallel to the weld, *N* corresponds to the sum of the nodal forces parallel to the weld and the nodal moments about the axis perpendicular to the weld direction.

As an idealized baseline reference, assuming temporarily that the vertical stress and shear stress vary proportionally (i.e., they are completely in-phase), the baseline equivalent stress frequency response function for the composite structure ${\Delta \sigma }_{e0}(\omega )$ can be obtained as Equation (15):

(15) $$\Delta \sigma_{e0}(\omega )=\sqrt{{\left(\;\Delta \sigma_{s}\right)}^{2}+\beta {\left(\Delta \tau_{s}\right)}^{2}}=\sqrt{Q_{1}^{2}+3Q_{2}^{2}}H_{d}(\omega )$$

Since *Q*_1_ and *Q*_2_ are both fundamental geometric parameters of the structure, this indicates that ${\Delta \sigma }_{e0}(\omega )$ exhibits a linear relationship with external loads.

However, this ideal proportional assumption rarely holds true in actual vehicle structures. It is crucial to clarify that while the structural dynamic system is assumed to be globally linear (as defined in Equations (4)–(6)), the local stress components at the weld are often non-proportional. Under broad-band random excitation (even unidirectional loads per IEC 61373), the spatial structural response is a superposition of multiple vibration modes. Because normal stress and shear stress are often dominated by different modal shapes, their frequency response functions naturally exhibit phase differences. This dynamic asynchrony leads to a typical non-proportional multiaxial stress state, which causes additional fatigue damage and necessitates the introduction of the correction parameter F(δ).

Under a uniaxial load, the load input itself exhibits no phase difference. However, the structure’s multi-directional modal vibration response can induce a multiaxial stress state at the weld. If a phase difference exists between the shear and normal stress responses, this represents vibration responses in different directions of the structure. The frequency response functions (FRF) for shear and normal stress can be obtained separately through harmonic response analysis. The FRF is a complex number containing information about the phase difference between the response and the load. Given identical loading conditions, the multiaxial frequency domain correction factor *F* and phase difference parameter δ can be defined based on the phase difference between the frequency response functions of the shear and normal structural stress (Equation (16)).

(16) $$\delta \left(\omega \right)=\arg\left({\left(\frac{H_{\sigma }\left(\omega \right)}{H_{\tau }\left(\omega \right)}\right)}_{img}/\left|\frac{H_{\sigma }\left(\omega \right)}{H_{\tau }\left(\omega \right)}\right|\right)$$

Based on the multiaxial equivalent structural stress calculation formula proposed by ASME standard, which provides the phase difference correction factor F(δ), we have formulated an approximate equation to represent the power spectrum of multiaxial equivalent structural stress under corrected non-proportional stress conditions. The calculation formula is as shown in Equation (17):

(17) $$H_{\sigma_{e}}(\omega )=\frac{1}{F\left(\delta (\omega )\right)}\sqrt{H_{\sigma }^{2}(\omega )+3H_{\tau }^{2}(\omega )}$$

Here, $H_{\sigma }(\omega )$ represents the normal structural stress frequency response function and $H_{\tau }(\omega )$ denotes the shear structural stress frequency response function, respectively; F(δ(ω)) is the undetermined correction parameter; and δ(ω) indicates the phase difference between shear and normal stress.

Taking plate thickness correction and load ratio correction into account, the power spectrum calculation formula for multiaxial equivalent structural stress is as Equation (18):

(18) $$H_{S_{e}}(\omega )=\frac{H_{e}(\omega )}{t^{2m}I(r)}$$

In the equation above, I(r) is a dimensionless function of the curvature ratio *r*, *m* = 3.6, and *t* denotes the plate thickness. At a specific frequency, I(r), *m*, and *t* are all constants. Therefore, the equivalent structural stress, like the structural stress itself, exhibits a linear transfer relationship with the applied load.

Since the IEC 61373 standard applies and calculates loads separately in the longitudinal, transverse, and vertical directions, if the spectral density of the uniaxial load input function X(t) is $S_{x}(\omega )$, and the spectral density of the output equivalent multiaxial structural stress response function $S_{S}(\omega )$ is $S_{S}(\omega )$, according to classical random vibration theory, the following relationship holds (Equation (19)):

(19) $$S_{Se}(\omega )={\left|H_{Se}(\omega )\right|}^{2}S_{x}(\omega )$$

The structural equivalent stress amplitude is a key parameter of the master S-N curve. Once the power spectral density (PSD) of the equivalent stress amplitude is obtained, the Dirlik method can be directly employed to estimate the probability density function (PDF) of the stress amplitude. In this evaluation framework, the Dirlik method is specifically adopted in this paper because, compared with the traditional narrowband Rayleigh approximation, it provides higher accuracy in the evaluation of broadband random processes. Moreover, in contrast to the time-domain rainflow counting method, the Dirlik method exhibits remarkable advantages in computational efficiency, which well satisfies the engineering demand for rapid fatigue life evaluation in the early stage of vehicle structural design [27].

Nevertheless, it should be emphasized that the Dirlik method is fundamentally based on the assumption that the underlying stress response is a stationary Gaussian random process. Although this assumption is highly consistent with the continuous broadband random excitation specified in the IEC 61373 standard, its prediction accuracy may deteriorate under highly non-Gaussian loading scenarios. Since the present study mainly focuses on stationary random vibrations, the Dirlik method achieves an optimal trade-off between computational accuracy and evaluation efficiency.

After obtaining the probability density function *P*(*S*) of the equivalent structural stress range based on the Dirlik method, the stress ranges and occurrence frequencies at each level per unit time can be determined [28]. Subsequently, applying Miner’s linear cumulative damage theory, the fatigue damage calculation formula for a specific point on the weld per unit time can be derived, as shown in Equation (20):

(20) $$E[D]=\sum_{i}\frac{n(S_{i})}{N(S_{i})}=\frac{S_{t}}{C_{d}^{1/h}}\int_{0}^{\infty }S_{Se}{\left(\omega \right)}^{1/h}P(S)dS$$

where $C_{d}$, *h* are parameters of the main S-N curve. If the shear stress reaches one-third or more of the normal stress during random vibration, its impact on weld fatigue must be considered.

**Figure 5:**
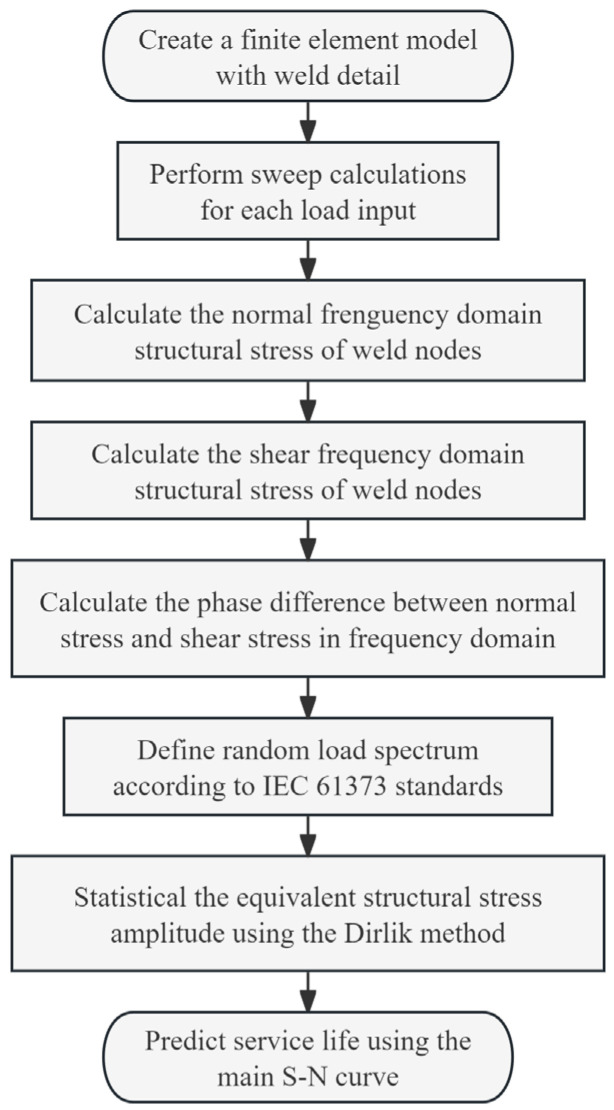
Flowchart of multiaxial weld fatigue assessment method based on IEC 61373 standard.

The technical workflow for evaluating structural random vibration fatigue of vehicle-mounted equipment based on IEC 61373 is illustrated in Figure 5 below:

### 3.2. Validation of the Method

**Figure 6:**
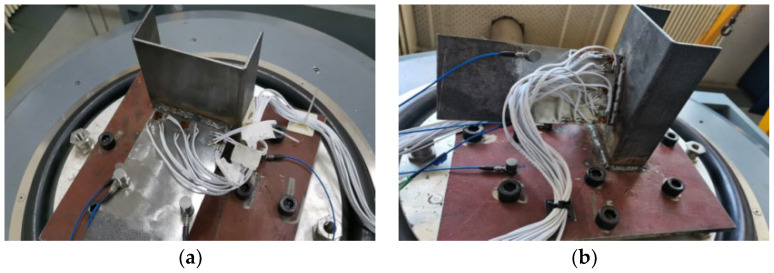
Welded specimens: (**a**) 0 degrees Specimen; (**b**) 30 degrees Specimen.

To validate the method’s effectiveness, vibration specimens were designed and subjected to resonance frequency, frequency response, random vibration, and phase angle tests using a random vibration testing apparatus. The tests analyzed the local stress distribution and modal vibration characteristics of the weld. Butt joints at 0 degrees and 30 degrees to the horizontal plane (Figure 6) were designed to investigate structural modal vibration and multiaxial stress states.

**Figure 7:**
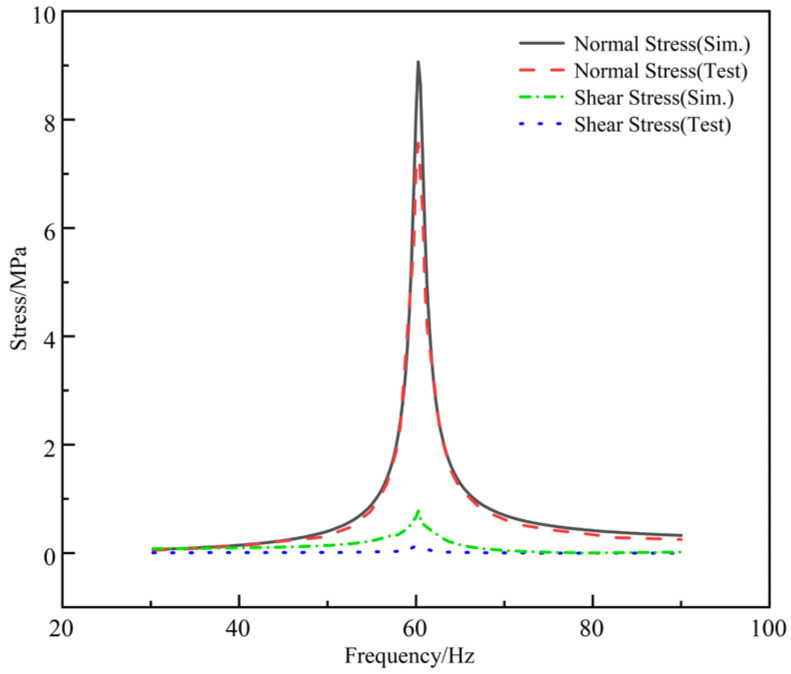
Test and simulation result.

Modal testing was conducted on specimens 0 degrees and 30 degrees (Specimen 1 and Specimen 2) to obtain the first-order constrained modal frequency and mode shape. The results are compared with calculations in Table 1 below:

The dynamic excitation was provided by an electromagnetic vibration testing system (Dongling Vibration Test Instrument Co., Ltd., Suzhou, China). To obtain the dynamic stress response of the 0 degrees specimen, triaxial strain gages (0°, 45°, 90°) manufactured by HBM (Darmstadt, Germany) were attached near the weld toe of the specimen. The data acquisition system (HBM, Darmstadt, Germany) utilized a Wheatstone bridge circuit with temperature compensation, and the strain gauges employed had a sensitivity coefficient of 2.10. All data were recorded using catman software from the same manufacturer Standard shunt calibration was performed under static conditions, and all measurement channels were activated to complete zeroing, thereby eliminating initial signal drift and ensuring the reliability of the test data. Following calibration, the specimen underwent fixed-frequency and swept-frequency vibration tests ranging from 30 Hz to 90 Hz. Test data indicates that the longitudinal shear stress in the weld is relatively small, and stress perpendicular to the weld direction remains within normal ranges (as shown in Figure 7). When the weld is perpendicular to the load direction, local stress is predominantly normal, with shear stress being negligible. Applying the same load in a finite element model using ANSYS software (Version 19.2, ANSYS, Inc., Canonsburg, PA, USA) for harmonic response analysis yielded the stress distribution at the weld. Comparison between simulation and test results is shown in Figure 7. Due to the distance between the test patch locations and the weld toe, the simulated peak values are slightly higher than the experimental values.

Since the laboratory is equipped only with a uniaxial vibration table and cannot perform multi-directional synchronous excitation, a flat test specimen inclined at a 30-degree angle to the horizontal plane was designed. A 54 Hz vertical single-frequency excitation was applied to induce local triaxial coupling effects between normal and shear stresses, thereby analyzing the structure’s triaxial stress state. Using pre-installed triaxial strain gages to continuously collect strain signals, the normal strain in the direction of the vertical weld was directly measured, and the shear strain parallel to the weld was calculated using Equation (21).

(21) $$\left\{\begin{array}{c} \gamma =2*\varepsilon_{45}-\varepsilon_{0}-\varepsilon_{90}\\ \sigma_{0}=E*\varepsilon_{0}\\ \tau =G*\gamma =\frac{E}{2\left(1+\eta \right)}*\gamma \end{array}\right.$$

Among these, γ represents shear strain; $\sigma_{0}$ denotes stress in the 0 degrees direction; *E* is the material’s elastic modulus; τ indicates shear stress; *G* represents the material’s shear modulus; and η denotes the material’s Poisson’s ratio.

**Figure 8:**
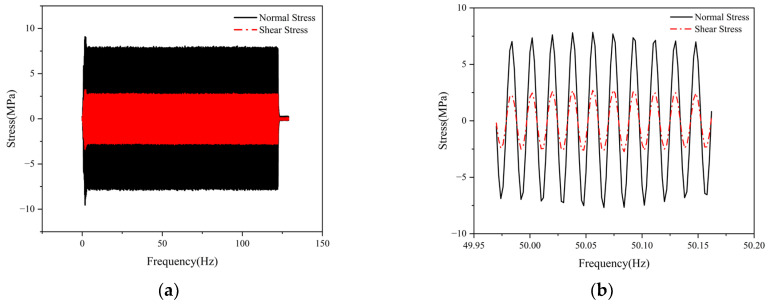
Vertical weld line stress diagram under 54Hz excitation: (**a**) Time history of normal stress and shear stress in vertical weld line under 54Hz excitation; (**b**) Enlarged detail.

As shown in Figure 8a, under single-frequency excitation, significant longitudinal shear stress develops at the weld of the 30 degrees specimen, with its amplitude exceeding 30% of the normal stress perpendicular to the weld direction—a value that cannot be ignored. The shear and normal stress in the time-domain signal exhibit essentially the same phase and distribute linearly proportional to each other. This indicates that even under uniaxial loading, where the weld modal vibration direction forms an angle with the loading direction, local shear stress is still induced at the weld. Stress distributions under other frequency excitations (e.g., 30, 50, and 60 Hz) are similar to those in Figure 8b, demonstrating that under these conditions, single-frequency load excitation can only induce linearly synchronized uniaxial stress states.

**Figure 9:**
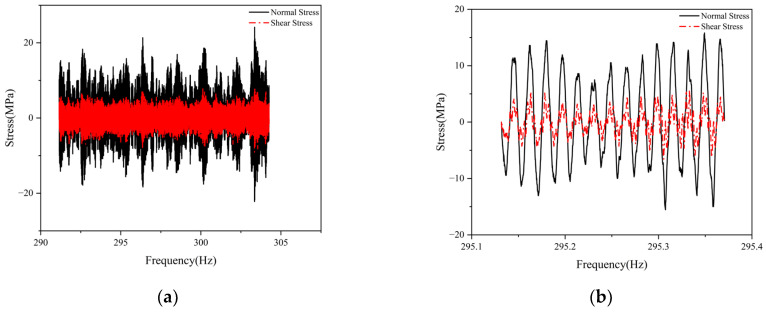
Test results of normal stress and shear stress at the end position of the weld: (**a**) Test diagram for normal stress and shear stress; (**b**) Enlarged detail.

To investigate the relationship between shear and normal stress in 30 degrees specimens under random loading, a vertical white noise random acceleration load ranging from 30 to 90 Hz was applied to their bases. Shear and normal stress were obtained through triaxial strain gauges, as shown in Figure 9a. Shear stress at the weld ends could reach up to 40% of the normal stress, indicating that shear stress significantly influence fatigue and cannot be neglected. Figure 9b shows that the two trends are similar but exhibit slight asynchrony, indicating that non-proportional effects must be considered.

**Figure 10:**
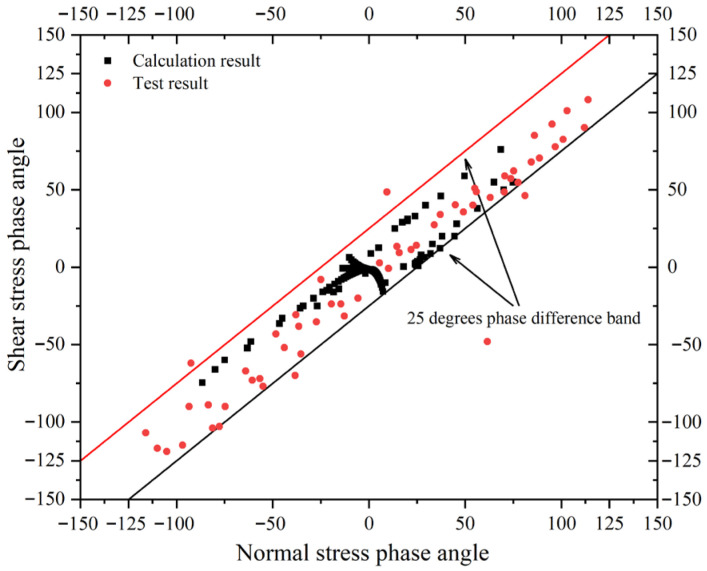
Phase angle comparison chart.

By applying Fourier transforms to the experimentally measured shear stress and normal stress, the distribution of their respective phases with frequency is obtained. The scatter plot of phase angles for normal and shear stress at the same frequency is shown in Figure 10. The results reveal that phase angles are predominantly concentrated in the central region, with relatively dense distributions along both the horizontal and vertical axes, while the remaining areas exhibit sparse distributions. The phase angle distribution obtained through the calculation of the stress frequency response function for normal and shear structures is essentially consistent with the distribution pattern of the measured data, and the phase difference between normal stress and shear stress is relatively small. Statistical data shows that all calculation results fall within the 25 degrees phase difference, with 73% within 15 degrees; the test results closely mirror this trend, with 92% within 25 degrees and 67% within 15 degrees. This indicates that the frequency-domain phase angle acquisition method proposed for this problem can effectively reflect the actual conditions.

**Figure 11:**
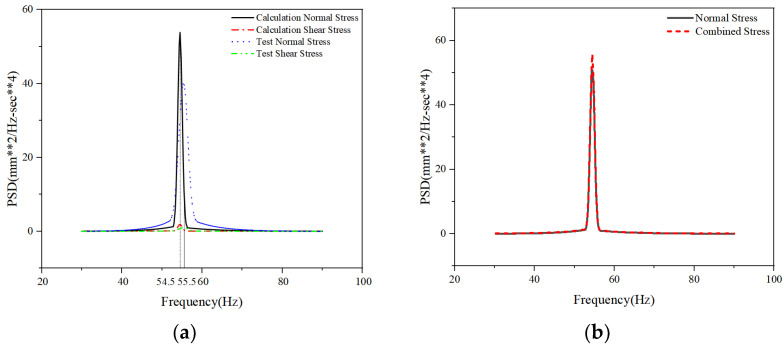
Comparison chart of normal stress and shear stress PSD for 30 degrees specimen: (**a**) Test PSD and Calculation PSD Diagram; (**b**) Calculation of normal stress and combined stress PSD Diagram. (In the y-axis label, the “**” symbol denotes exponentiation, where “mm^2^” represents square millimeters and “sec**4” represents seconds to the fourth power).

As shown in Figure 11a, and as shown in the power spectrum distribution plots for normal and shear stresses, near the structural modal frequency (e.g., 55 Hz), a slight discrepancy exists between the power spectrum responses (PSD) of the simulation model and the actual structure due to a certain deviation in their first-order bending modes, resulting in a slight deviation in the dominant frequency positions of their PSDs. Quantitatively, the simulated resonance frequency deviates from the experimental result by approximately 1 Hz, while the peak amplitude of the simulated PSD (approximately 53.85) is about 25.5% higher than the measured value (approximately 40.1). This discrepancy primarily stems from the inherent simplifications in the finite element boundary conditions, which slightly alter the structure’s first-order bending stiffness and modal damping. Regarding the impact of this discrepancy on the final fatigue life calculation, since fatigue damage is exponentially related to stress amplitude, an overestimation of the PSD peak would theoretically result in a conservative life prediction. However, given that the frequency-domain method proposed in this paper employs the Dirlik method to statistically integrate the structural stress response across the entire frequency band—rather than relying on a single, specific resonance peak—this broadband integration mechanism effectively “smooths out” the PSD amplitude deviations in localized frequency bands. Consequently, calculation errors in cumulative damage are effectively suppressed, ensuring that the final multiaxial equivalent fatigue life prediction remains robustly within the engineering-acceptable tolerance range.

To visually assess the theoretical contribution of shear stress to the total fatigue damage, Figure 11b further compares the PSD of uniaxial stress with the PSD of equivalent multiaxial stress that accounts for shear stress. As clearly shown in the figure, after introducing shear stress components, both the PSD peak and the band energy of the comprehensive structural stress response exhibit a certain increase compared to the uniaxial state. This stress amplitude amplification effect in the frequency domain directly provides the underlying mathematical and mechanical foundation for the calculation results in the subsequent multiaxial evaluation, where the fatigue life is significantly lower than that of the uniaxial evaluation.

Furthermore, deviations in the high-frequency range are primarily due to the limitations of the IEC 61373 standard. The power spectral density curve specified in the IEC 61373 standard is an envelope spectrum designed to conservatively account for extreme operating conditions. This broadband envelope treatment tends to overestimate the actual vibration energy input at high frequencies, leading to a theoretical “over-excitation” phenomenon in simulations. Therefore, the high-frequency deviation reflects the highly conservative nature of the standard load spectrum.

**Figure 12:**
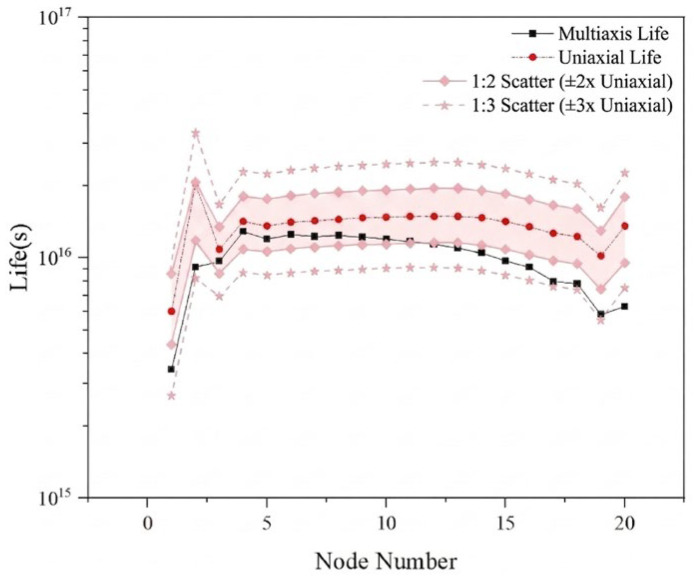
Comparison chart of uniaxial and multiaxial service life of 30 degrees specimens.

As shown in Figure 12, to visually evaluate the discrepancies between uniaxial and multiaxial fatigue life predictions, 1:2 and 1:3 error bands were introduced with the uniaxial fatigue life as the reference. An analysis of the data distribution shows that the multiaxial fatigue life, when shear stress is considered, is generally lower than the uniaxial predicted life. Moreover, from the viewpoint of engineering tolerance evaluation, the multiaxial life results of all 20 nodes along the same weld seam fall entirely within the 1:3 error band, and the vast majority of nodes lie within the stricter 1:2 error band. This result demonstrates that although the traditional uniaxial evaluation method is somewhat non-conservative, the prediction deviation between multiaxial and uniaxial approaches generally remains within the acceptable error range for the conventional fatigue life assessment of rail transit equipment.

## 4. Fatigue Assessment of Vehicle-Mounted Electrical Cabinet Based on IEC 61373 Standard

### 4.1. Multiaxial Fatigue Assessment of Electrical Cabinet Frame

The electrical cabinet frame serves as the critical structural component for installing electrical equipment in rail vehicles. Mounted on the vehicle floor, it primarily bears vibration loads transmitted from the track. To validate the reliability of the proposed method for fatigue assessment of vehicle-mounted equipment, a vehicle-mounted electrical cabinet structure was specifically designed.

**Figure 13:**
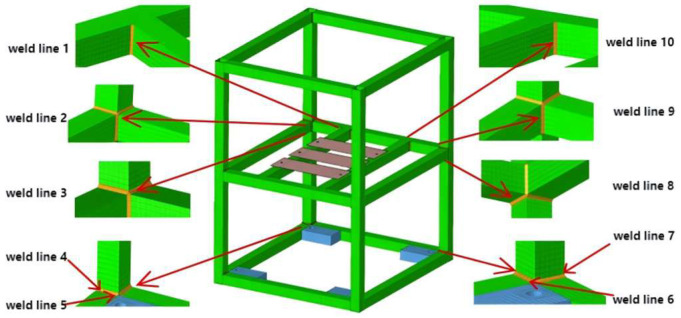
Distribution map of 10 weld lines.

A finite element model of this structure was established, comprising the base, frame, and welded plates, all fabricated from aluminum alloy. The model meticulously accounts for the influence of weld geometry on local stress concentration. Shell elements were employed for the frame and welded plates, while hexahedral meshes were used for the base. Frame and center beam connections were simulated using ramp weld lines, with weld-area elements modeled as variable thickness ($1/\sqrt{2}$ times plate thickness). Based on structural configuration and modal characteristics, calculations focused on 10 critical weld lines as shown in Figure 13.

Based on Class A loads specified in the IEC 61373 standard, random vibration fatigue life simulations were performed on 10 welds in the frame structure of an electrical cabinet. The simulation frequency range was 5–150 Hz, with a frequency interval of 0.5 Hz. The S-N curve representing the material’s fatigue characteristics was selected for aluminum alloy, and the standard deviation was set to the median value. Identical loads were applied to the 10 welds in the X, Y, and Z directions, respectively. A 5-h fatigue simulation was conducted independently for each direction. Finally, the damage results from the three uniaxial simulations were combined, and a comparative analysis was performed between uniaxial and multiaxial conditions; evaluation results are shown in Table 2.

**Figure 14:**
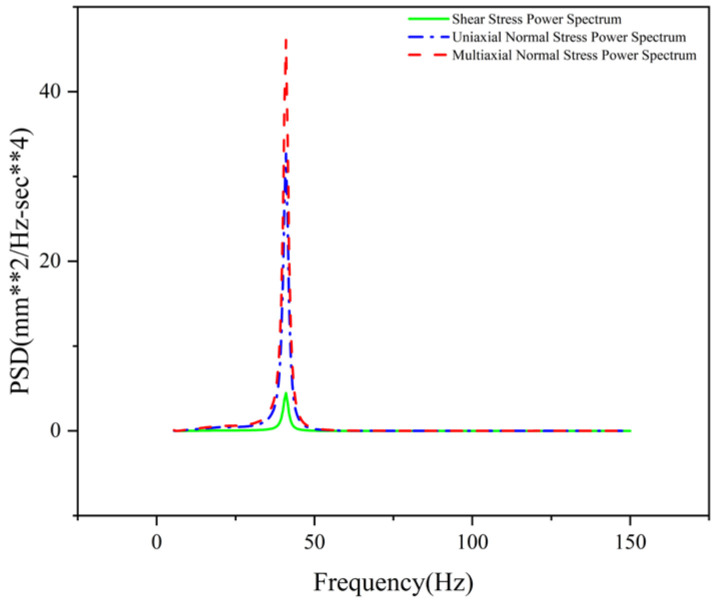
Comparison chart of uniaxial and multiaxial power spectrum of weld line 2. (In the y-axis label, the “**” symbol denotes exponentiation, where “mm^2^” represents square millimeters and “sec**4” represents seconds to the fourth power).

Comparing the equivalent fatigue life results between uniaxial and multiaxial evaluations for this structure reveals that multiaxial fatigue assessment generally yields lower life values than uniaxial results. This indicates potential safety concerns with the uniaxial method due to its neglect of shear stress, whereas the multiaxial random vibration fatigue assessment method considers factors more comprehensively. The deviation range between uniaxial and multiaxial evaluation results varies across different weld locations (between 4.20% and 88.35%), correlating with local structural deformation. Weld line 2, a vertical weld in the frame’s mid-section, experiences minimal shear stress under Y-direction loading, resulting in a low shear stress power spectrum (as shown in Figure 14). In contrast, fatigue weak points at weld line 10 are primarily influenced by shear stress, where the multiaxial fatigue life is significantly lower than the uniaxial result. This demonstrates that the multiaxial equivalent method provides a more comprehensive assessment, yielding more reliable fatigue evaluation outcomes.

The discrepancies in fatigue life predictions between the single-axis and multi-axis methods listed in Table 2 vary significantly across different welds (ranging from 4.20% to 88.35%). The root cause of these discrepancies is intrinsically linked to the complex dynamic mechanical response of the electrical cabinet frame under random vibration, as well as its spatial geometric characteristics.

**Figure 15:**
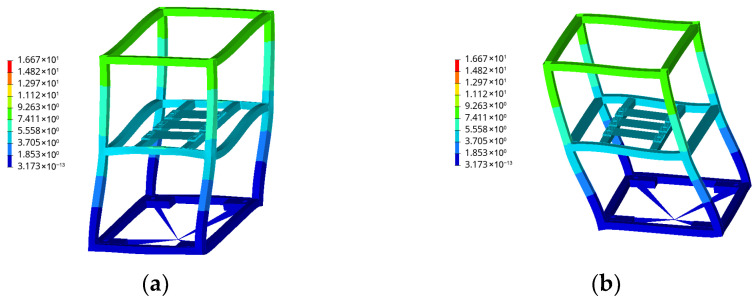

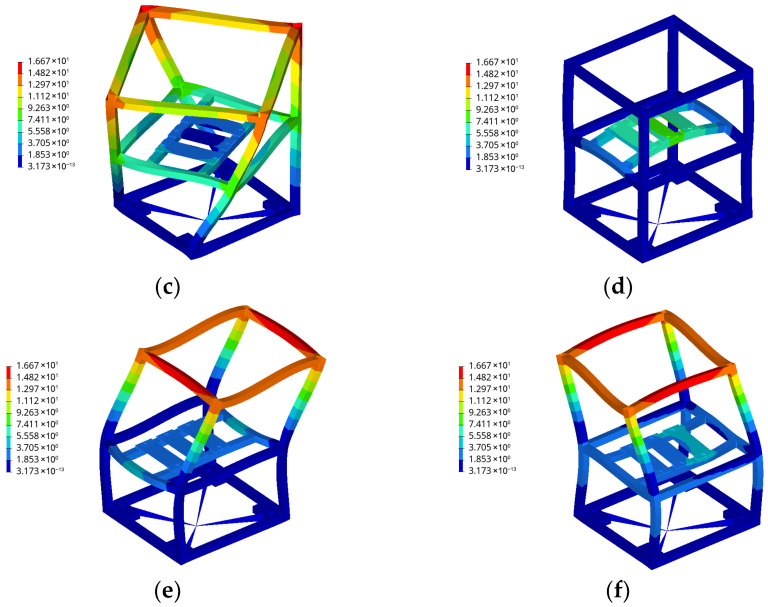
Typical modal deformation shapes of electrical cabinet frames within the excitation frequency band: (**a**) First-order mode; (**b**) Second-order mode; (**c**) Third-order mode; (**d**) 4th-order mode; (**e**) 5th-order mode; (**f**) 6th-order mode.

As shown in Figure 15, the first six modal orders of this frame are extracted in this study. The first (40.744 Hz) and second (40.757 Hz) modal orders of the structure manifest as overall bending deformation along the horizontal axis. This macroscopic bending primarily induces tensile stresses on the symmetrical frame columns and beams (such as welds 1 and 2). Therefore, for these connection areas dominated by tensile stresses, the uniaxial method provides a relatively accurate assessment, and Table 2 shows that the reduction in service life after considering multiaxial effects is small.

In stark contrast, the structure exhibits a strong global torsional mode at the 3rd order (73.315 Hz), while localized bending deformation of the intermediate support beams occurs at the 4th order (83.970 Hz) and higher orders. Under broad-band random excitation, this strong torsional response subjects the asymmetric corner joints, bottom constraints, and multi-way intersections (such as welds 3, 8, and 10) to bear extreme out-of-plane shear stresses, resulting in severe local tensile-shear coupling. Since traditional uniaxial evaluation methods completely ignore this critical shear stress damage caused by torsional modes, they dangerously overestimate the durability of these specific corner joints, leading to a significant evaluation deviation of up to 88.35% as shown in Table 2.

The aforementioned modal-based mechanical attribution analysis highlights the practical engineering value of the method presented in this paper: for vehicle equipment structures that inherently exhibit significant torsional responses or complex local deformations, conventional uniaxial evaluations pose significant safety risks. In actual engineering design, not only must multiaxial frequency-domain methods be employed for life correction, but designers should also prioritize local structural reinforcement at the corner junctions of the enclosure based on the stress distribution of higher-order torsional modes.

### 4.2. Experimental Verification of Multiaxial Fatigue Assessment for Vehicle-Mounted Electrical Cabinet

**Figure 16:**
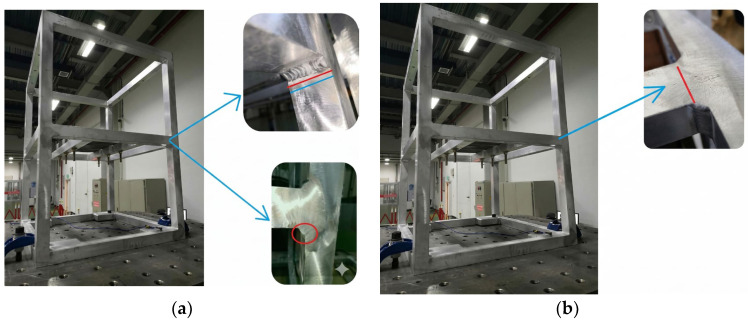
Comparison chart of uniaxial and multiaxial power spectrum of weld line 2: (**a**) Failure diagram 1; (**b**) Failure diagram 2.

To further validate the reliability of this method for fatigue assessment of vehicle-mounted equipment in rail vehicles, two electrical cabinet samples were subjected to random vibration testing. To accelerate testing and reduce costs, the loads were amplified. Calculations based on the multiaxial fatigue method revealed that lateral random loads significantly impact service life. Therefore, the multiaxial fatigue method proposed in this paper was applied to fatigue calculations for critical welds in electrical cabinets. The Class A load from IEC 61373 was amplified by factors of 2, 3, and 4. When the load was increased to 4 times the original value, the fatigue life of the critical weld reached 6.32 h (as shown in Table 3), indicating that fatigue failure could potentially occur after approximately 6 h of excitation. Subsequent testing with two 4x load cycles resulted in fatigue failure at the weld, with the specific location shown in Figure 16.

Under a transverse random load four times the IEC 61373 Class A standard, the first specimen exhibited a distinct crack in the weld at the midpoint of the bracket after 5.85 h; the second specimen showed a microcrack at the same location after 4.93 h. The average specimen life was 5.39 h, with cracks consistently appearing at the weld with the lowest calculated life. This demonstrates that the method can accurately identify fatigue weak points, and multiaxial life assessment results are closer to experimental values than uniaxial assessments.

## 5. Conclusions

Based on IEC 61373, this paper proposes a novel method for evaluating multiaxial random vibration fatigue in vehicle-mounted equipment of rail vehicles. This approach accounts for the effect of multiaxial stress states in welds on fatigue life. Through computational analysis and experimental comparison, the primary findings are as follows:

1. Simulation and test results of the prototype indicate that when the direction of modal vibration in the weld joint forms an angle with the loading direction, shear stress may develop locally within the weld. The magnitude of shear stress can exceed 30% of the normal stress, necessitating their consideration during fatigue assessment.
2. The multiaxial fatigue assessment method proposed in this paper can account for the influence of shear stress on fatigue life while also considering the phase difference between weld shear forces and normal stress caused by modal vibrations of the structure. Simulation and experimental results demonstrate that even under uniaxial random loading, a multiaxial stress state may develop locally within the weld, particularly when the structural modal direction forms an angle with the loading direction.
3. Overall, the results of this simulation generally align with the initial theoretical expectations, although some local deviations are observed. The findings confirm that the impact of multi-axis combined damage on weld life is greater than that of single-axis damage, thereby validating the assessment assumptions presented in this paper. However, the results also indicate significant location-dependent variations among welds at different locations on the same structure, with considerable fluctuations in the magnitude of deviations across different sites. The results clearly demonstrate that when conducting life assessments for complex structures, traditional uniaxial calculation methods carry the risk of underestimating structural damage; therefore, a multiaxial fatigue damage synthesis method must be adopted to obtain safer and more accurate life predictions.
4. The multi-axis fatigue method proposed in this paper enables rapid assessment of random vibration fatigue in rail vehicle welded structures, shifting the design paradigm from a conservative, uniaxial load-dominated approach to a targeted multi-axis loading approach. By combining multiaxial structural stress methods with the IEC 61373 standard, this study transforms complex fatigue theory into a highly practical engineering tool. It enables designers to identify and address critical fatigue vulnerabilities through computational means, thereby reducing reliance on repeated and costly physical vibration test bench trials, lowering development costs, and shortening product design cycles. Consequently, this method provides a practical and cost-effective technology for rail vehicle manufacturing, offering significant engineering value.

## Figures and Tables

**Table 1:** Comparison of modal frequency and calculation.

Specimen Number	Specimen Deformation Diagram	Test Modal Frequency/Hz	Calculate Modal Frequency/Hz	Error/%
Specimen 1	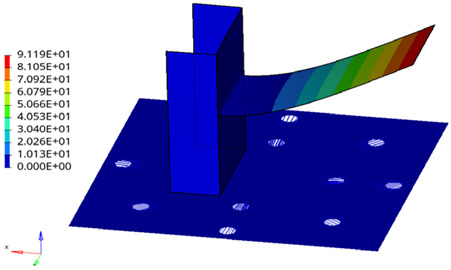	58.9	60.35	2.4
Specimen 2	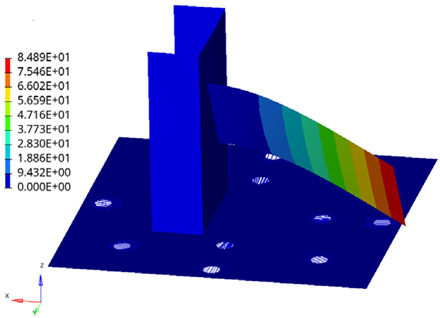	54.4	55.54	2.1

**Table 2:** Comparison of damage in 10 critical weld lines.

Weld Line	Vibration Duration in Each Direction	Uniaxial Composite Damage	Multiaxial Composite Damage	Uniaxial Equivalent Life/h	Multiaxial Equivalent Life/h	Reduction in Life/%
1	X: 5 h Y: 5 h Z: 5 h	9.62 × 10^−4^	1.05 × 10^−3^	1.56 × 10^4^	1.43 × 10^4^	8.57
2		7.88 × 10^−3^	8.34 × 10^−3^	2.07 × 10^3^	1.90 × 10^3^	5.54
3		1.65 × 10^−3^	5.03 × 10^−3^	9.09 × 10^3^	2.98 × 10^3^	67.21
4		6.09 × 10^−3^	7.83 × 10^−3^	2.46 × 10^3^	1.92 × 10^3^	22.15
5		6.65 × 10^−4^	1.90 × 10^−3^	2.26 × 10^4^	7.91 × 10^3^	64.94
6		3.53 × 10^−3^	6.44 × 10^−3^	4.25 × 10^3^	2.33 × 10^3^	45.21
7		1.31 × 10^−3^	2.50 × 10^−3^	1.15 × 10^4^	6.01 × 10^3^	47.59
8		1.65 × 10^−3^	5.03 × 10^−3^	9.09 × 10^3^	2.98 × 10^3^	67.21
9		7.56 × 10^−3^	7.89 × 10^−3^	2.18 × 10^3^	1.99 × 10^3^	4.20
10		1.76 × 10^−4^	1.51 × 10^−3^	8.53 × 10^4^	9.94 × 10^3^	88.35

**Table 3:** Comparison of weld line 2 life.

	4x Load Test Result/h	1x Load Minimum Life/h	2x Load Minimum Life/h	3x Load Minimum Life/h	4x Load Minimum Life/h	Test Error/%
Uniaxis	5.39	2.72 × 10^3^	2.23 × 10^2^	5.16 × 10^1^	7.32 × 10^00^	26.36
Multiaxis		2.68 × 10^3^	2.19 × 10^2^	5.08 × 10^1^	6.32 × 10^00^	14.66

## Data Availability

The original contributions presented in this study are included in the article. Further inquiries can be directed to the corresponding author.
